# Links of Adversity in Childhood With Mental and Physical Health Outcomes: A Systematic Review of Longitudinal Mediating and Moderating Mechanisms

**DOI:** 10.1177/15248380221075087

**Published:** 2022-02-28

**Authors:** George K. Hales, Zeliha E. Saribaz, Agata Debowska, Richard Rowe

**Affiliations:** 1 Department of Psychology, 7315 University of Sheffield, Sheffield, UK; 2 7315 SWPS University of Social Sciences and Humanities, Poznan, Poland

**Keywords:** alcohol and drugs, child abuse, violence exposure, youth violence

## Abstract

Adverse childhood experiences (ACEs) have been associated with causes of early death, addiction, mental illness, and poor health. However, studies investigating underlying mechanisms often rely on cross-sectional data or inappropriate study designs. To prevent the negative sequelae associated with ACEs, it is imperative to understand the mechanisms underlying the prospective relationship. The aim of this present review was to provide a synthesis and critical evaluation of the literature regarding the mechanisms underlying this relationship. A search in SCOPUS, MedLine via Ovid, PsycINFO via Ovid, and Web of Science was performed. Studies that utilised a prospective design assessing ACEs in childhood or adolescence, outcomes in adulthood, and analysed either a mediating or moderating relationship were included, unless the study relied on informant report or official records to assess childhood maltreatment types of ACEs. Twenty-two studies examining a longitudinal mediation or moderation were included in a systematic review. A review of the studies found links to psychopathology, delinquent and problem behaviours, poor physical health, and poor socioeconomic outcomes. A clear image of underlying mechanisms is not forthcoming due to (a) poor study design in relation to assessing longitudinal mechanisms, and (b) heterogeneity in the adversities, mechanisms, and outcomes assessed. Based on the review, several gaps and limitations are highlighted and discussed.

Adverse childhood experiences (ACEs) are the focus of much research. Consistently ACEs have been found to impact childhood development and psychosocial functioning. Efforts to understand this relationship are marred by methodological difficulties and inadequacies such as an overreliance on officially documented cases of abuse and cross-sectional study design. Officially documented cases of abuse only scratch the surface of the true prevalence of abuse and might be prone to biases. Cross-sectional study design is a sub-optimal methodology when used to investigate underlying mechanisms in a longitudinal relationship. To better understand what drives the purported relationship between ACEs and psychosocial functioning, this review will focus on studies that utilise prospective self-report designs to explore mediating and moderating variables.

ACEs involve a wide range of inter-correlated factors including child maltreatment (e.g. physical abuse, sexual abuse, emotional abuse and neglect) and household dysfunction (e.g. parent divorce, parental mental illness and parental substance abuse) (Felitti et al., 1998). Some studies have used factor analysis to formally examine the underlying structure. While ACEs broadly lead to similar outcomes, there are a number of different ways ACEs can be conceptualised. There is some empirical evidence that child maltreatment and household dysfunction variables can be separated although findings are mixed. For instance, an exploratory analysis found that a 3-factor solution (household dysfunction, physical/emotional abuse and sexual abuse) best fit the data collected using the Behavioural Risk Factor Surveillance System survey (Ford et al., 2014). Notably, the three factors correlated highly with one another, possibly indicating a higher order factor of ACEs. Another analysis found that a 2-factor solution best fit 10 ACEs among a low-income sample of women who received home visiting services, but when six additional adversities were added, a 4-factor solution provided a better fit, although the eigenvalue for the fourth factor was lower than 1 which might indicate limited variance is explained by this factor (Mersky et al., 2017). The four factors corresponded to interpersonal victimisation (including maltreatment and household dysfunction items), emotional and physical neglect, extreme poverty, and family loss or separation. A similar study found that a 2-factor solution was the best fit even where additional adversities were included, wherein peer victimisation experiences were grouped with child maltreatment items (Afifi et al., 2020). It may be that child maltreatment and household dysfunction are distinct subtypes of ACEs in conventional models, but additional ACEs might lead to different patterns.

There is a large evidence base showing that child abuse and neglect predict numerous negative adult outcomes including poor mental health, substance abuse, risky sexual behaviour, obesity and delinquency (see Gilbert et al., 2009 for a review). A range of evidence shows that specific household dysfunction variables such as parental incarceration are related to negative effects in childhood including antisocial behaviour (e.g. Murray et al., 2012). Broadly defined household dysfunction is associated with a range of negative outcomes (Andersen, 2021). However, some household dysfunction items such as familial financial problems, death of a parent/close relative and separation from family have received less research attention regarding adult outcomes (see Hughes et al., 2017). Comparative research has demonstrated that child maltreatment items are more salient than household dysfunction items at predicting mental health issues in early adulthood (Negriff, 2020). Child maltreatment is common in the UK; 24.5% of young adults retrospectively report being a victim of at least one type of maltreatment by their parents (Radford et al., 2011). A prevalence study in the USA found that household dysfunction is more prevalent than child maltreatment (Finkelhor et al., 2015). The same study also proposed adding other variables to measures of ACEs, including low socioeconomic status, high peer victimisation, high peer social isolation and exposure to community violence which were purported to have improved the measure. There is appetite among researchers to iterate ACE measures by including more childhood adversities, and so this systematic review will use a broad definition of ACEs. Finkelhor et al. (2015) found that family mental illness (32.5%) was the most prevalent of the ACEs measured, with high peer social isolation (22.5%), parental divorce/separation (21.3%) and physical neglect (15.9%) also relatively prevalent; Radford et al. (2013) found that exposures to community violence (66.5%), peer victimisation (63.2%) and physical violence from a non-caregiver (55.5%) were the most commonly reported ACEs. Females reported an increased prevalence of lifetime sexual and intimate partner violence, whereas males reported an increased prevalence of lifetime violent victimisation.

It is thought that exposure to multiple types of adversity confers a more potent effect on the individual, resulting in a higher risk of outcomes, or worse outcomes (see Felitti et al., 1998; Finkelhor et al., 2011). Typically, studying ACEs takes the form of assessing the cumulative risk of ACEs, a putative relationship between a summed score of adversities and subsequent outcomes. Indeed, the basis for this approach is that several research articles report co-occurrences between ACEs (see Cecil et al., 2017; Finkelhor et al., 2007, 2009) which confers a greater risk of negative sequelae (Cyr et al., 2014; Hunt et al., 2017; Merrick et al., 2017). Subsequent systematic reviews have generally concurred that exposure to four or more types of ACEs reflects a high risk of negative outcomes. For instance, one meta-analysis of studies that included a risk estimate for individuals exposed to four or more ACEs found that such exposure confers a high risk of several outcomes including suicide attempts, substance abuse or problematic alcohol use and interpersonal violence (Hughes et al., 2017). Notably, these outcomes would constitute an adverse environment for rearing children, perhaps demonstrating evidence of a cycle of adversity. A systematic review of studies assessing risk factors for involvement in weapon-related crime in young people in the UK found that ACEs and prior victimisations were risk factors (Haylock et al., 2020). Further, a systematic review of studies relating ACEs to sleep disorders found that the strength of the putative association increased with the number and severity of ACEs (Kajeepeta et al., 2015). While these systematic reviews have outlined the magnitude of risk conferred by ACEs on negative outcomes in adolescence and adulthood, none reported on plausible mechanisms underlying the longitudinal relationship. One systematic review explored how aspects of the home environment and parenting behaviours might mediate the relationship between ACEs and cognitive development (Guinosso et al., 2016). However, this study focused on an outcome in childhood, thus limiting the scope of understanding longitudinal impacts. Another systematic review focused on mechanisms that explain the relationship between ACEs and obesity in adulthood, finding that commonly cited mechanisms included social disruption, health behaviours and chronic stress response (Wiss & Brewerton, 2020). One weakness common to all these systematic reviews is that cross-sectional studies frequently accounted for a substantial proportion of included studies. Cross-sectional study design is a sub-optimal approach for studying time-dependent relationships, meaning that the current understanding of how ACEs affect longitudinal outcomes should be tempered.

## Studying underlying mechanisms

There is growing interest in investigating the mechanisms underlying the relationship between childhood adversity and distal outcomes in adulthood. A number of theoretical frameworks invoke a role of intervening variables (e.g. Grych et al., 2015), which can be tested using mediation models. These models are most usefully applied where there are theoretical mechanisms linking ACEs to outcomes. There are also methodological obstacles to consider when investigating potential mechanisms influencing the putative relationship. One such obstacle is that ideal study design must be balanced with ethical concerns about the welfare of children at risk; purposefully exposing children to ACEs as experimental manipulation would be unethical. Much knowledge regarding the impact of ACEs has relied on cross-sectional studies and retrospective recall. Indeed, the original ACEs dataset relied on cross-sectional design (see Felitti et al., 1998). When assessing mediation, temporal ordering of variance is an important consideration. A reliance on cross-sectional data to infer mediational processes could be highly misleading because mediational models imply change over time, but cross-sectional data obfuscates the time-lagged effects of a purported risk factor or mediator. Additionally, cross-sectional designs fail to consider whether the putative relationship between adversity and negative outcomes could be explained by confounding variables (Jaffee et al., 2012). Collecting prospective data in a sequential design minimises uncertainty concerning temporal biases affecting observed results.

A key issue regarding data collection for childhood adversities is reliability. One way to test the reliability of different data collection methods is to compare agreement between methods. A recent meta-analysis tested the concordance between prospectively and retrospectively collected child maltreatment data (Baldwin et al., 2019). Agreement was poor for child maltreatment but substantially concordant for childhood separation from parents. Self-report in adolescence has been found to indicate the highest prevalence of ACEs when compared to caregiver reports and retrospective recall (Naicker et al., 2017); findings elsewhere indicate incongruence between reports of physical abuse collected concurrently during adolescence and retrospectively at age 30 (White et al., 2007). However, it should be noted that we do not know the extent to which individuals may overreport or misrepresent their experiences of adversity, especially when accounts rely on retrospective recall alone (see Widom et al., 2004).

Alternative methods include court-substantiated cases or informant reports. Research in the UK has estimated that most child maltreatment victims are not officially documented, as rates of child maltreatment measured by a combination of self-report and parent informants are between 7 and 17 times more common than officially documented cases (Radford et al., 2013). A similar finding supports this general assertion with a Portuguese sample (Pinto & Maia, 2013). While substantiated child maltreatment data enables researchers to study verified cases, or the most severe cases (Shaffer et al., 2008), researchers interested in any occurrence of child maltreatment might favour prospective self-report or informant report instead. Further, children from Black and Latin American populations in the USA are at an increased risk of involvement with child protection services and placement into foster care (Putnam-Hornstein et al., 2013). Findings from the UK indicate that the putative role of ethnicity in child protection services involvement may need to be considered in conjunction with neighbourhood deprivation (Bywaters et al., 2017). It is unclear why such biases might exist. One potential explanation is that social workers might expect more maltreatment to be present in troubled homes and formally report more alleged cases that meet their expectations (Debowska et al., 2020). Nevertheless, it is anticipated that while not immune to biases, self- or informant-report in representative samples might assuage some of these weaknesses of substantiated child maltreatment data.

Informants such as parents and teachers may provide reliable data regarding ACEs in young children. There are some concerns regarding underreporting of child maltreatment when using informant-report (Fisher et al., 2011). Additionally, insights from the E-Risk longitudinal dataset found that the agreement between retrospective self-report and prospective informant report of child maltreatment is only slight (Newbury et al., 2018). The World Health Organisation (Meinck et al., 2016) recommends that children and young people aged 10-17 should be the target sample to collect self-reported child maltreatment data. Several self-report measures have been designed specifically for this age range, such as the Juvenile Victimisation Questionnaire (JVQ), which demonstrates adequate psychometric properties (see Finkelhor et al., 2005). It is assumed that children who can self-report child maltreatment are of appropriate maturity to also report household dysfunction and other adversities such as bullying, although household dysfunction may just as easily be reported by informants. Clinical interviews can be used to improve accessibility for younger children or participants with impairments (Finkelhor et al., 2005), which broadens the reach of self-report data. Despite adequate measures being available to collect self-report data, data may still be unreliable due to the immaturity or cognitive impairments of participants, erroneous memories or refusal to report adverse experiences to research teams. Therefore, informant report is a useful component of ACEs research.

Mediation is an important component for inferring the role of indirect relationships (Kenny, 2008), especially in the absence of randomised controlled trials. Moderation is also an important tool, particularly to identify if the relationship between ACEs and varies according to the level of a third variable (Baron & Kenny, 1986) such as sex, ethnicity, genetic polymorphisms or socioeconomic status. Both analytic methods are important and will be reviewed in tandem. For the purposes of this review, a cross-lagged panel model (CLPM) is highlighted as a minimally appropriate way to study putative longitudinal mediation. The CLPM involves deliberately staggering measurements of independent variable, mediator and dependent variable (X, M and Y) through sequential design (see Preacher, 2015 for a discussion of mediation models using longitudinal data). This requires at least three time points, corresponding to time lags in which the independent variable and mediator can affect the dependent variable. This is important because mediation is essentially a longitudinal process, so estimating mediation using cross-sectional data can be misleading (see Maxwell & Cole, 2007). Reducing this model to two phases introduces greater uncertainty as to the impact of the mediator on the direct relationship because only a partial effect of time can be observed (Mitchell & Maxwell, 2013). Additionally, deliberately staggering measurements raises an issue regarding the extent to which a variable is stable over time. If an outcome variable is relatively stable over the time of measurement, direct or indirect relationships could be an artefact of pre-existing variance. Indeed, other authors have suggested different models such as random intercepts cross-lagged panel model (RI-CLPM), autoregressive latent trajectory model with structured residuals or dual change score model as more appropriate when a variable is time-invariant (see Hamaker et al., 2015; Mund & Nestler, 2019). Using the correct model to test the putative mechanism is of utmost importance to ensure claims being made are accurate (Orth et al., 2021).

It seems likely from the evidence laid out above that each method of data collection has different advantages and disadvantages, and often data from different sources identify different groups of individuals (Baldwin et al., 2019). In addition, prospective self- or informant-report data collection methods among a representative sample eschews potential biases associated with court substantiated or child protection services data. Prospective self- or informant-report data relies less on life scripts and memory biases than retrospective data (see Widom et al., 2004). Moreover, a CLPM is coherent with repeated measures self- or informant-report designs. To allow for meaningful comparisons between the studies, this present review will test the distal effects of ACEs using prospective self-report data collected among children and adolescents to assess ACEs where feasible but will allow household dysfunction variables to be measured by caregiver reports and other informants. From the discussion above, it seems that child maltreatment data varies substantially based on data collection method, whereas there is less evidence that household dysfunction variables will vary based on the method of data collection.

## The current study

This present systematic review aims to synthesise research using longitudinal designs to examine the impact of mediators and moderators in the relationship between ACEs and negative outcomes. The present systematic review will include studies using prospective self-report data of ACEs and informant report of household dysfunction variables. This approach has been taken because of the underreporting of child maltreatment by official records (Radford et al., 2013; Shaffer et al., 2008) and reliability concerns of retrospective data (Widom et al., 2004). Additionally, the use of substantiated cases of child maltreatment does not conform with the purpose of assessing prospective studies in this review. The inclusion of studies that use informant report for household dysfunction variables is made on the assumption that such biases do not affect judgements regarding household dysfunction variables and the lack of evidence to contradict this assumption. Anticipating a low number of studies, the systematic review will have a broad focus of outcomes including mental health, physical health and life adjustment outcomes. This present systematic review is distinguished by primarily focusing on mediation and moderation analyses which use prospective data, which is of fundamental importance to investigating time-dependent relationships.

## Method

### Search strategy

The systematic review protocol was registered on PROSPERO CRD42020169259.

Empirical research included in this review used prospective data to examine mediating or moderating pathways between adversities experienced in childhood and outcomes in adulthood. Studies included must have collected data on multiple ACEs prior to the age of 19 and followed participants into adulthood to assess physical, mental, social, behavioural, cognitive or economic outcomes. ACEs was defined as the measurement of two or more exposures to ACEs previously defined by Felitti et al., 1998 and revised by Finkelhor et al., 2015. Using these definitions, several ACEs were focused on in this review (see Table 1). Studies that enquired about ACEs exposure ever during childhood or in a temporally specified time (e.g. in the last 12 months) were included. There must have been a minimum of two data collection time points for a study to be included, where ACEs and outcome variables were measured in temporal order. Studies that relied on court-substantiated cases of child maltreatment or caregiver reports of child maltreatment were excluded. Informant reports of household dysfunction variables were included.

**Table 1.** Sub-categories of adverse childhood experiences used in this review.

Childhood Maltreatment	Household Dysfunction	Other
Physical abuse	Household mental illness	Peer victimisation/bullying
Sexual abuse	Household criminality	Peer rejection
Emotional abuse	Household alcohol abuse	Community violence
Neglect	Household substance use	Witnessing crime
Harsh punishment	Domestic violence/abuse	Criminal victimisation
Low caregiver warmth	Financial hardship	Multiple hospitalisations
	Parental divorce/separation	Chronic illness
	Death of family member	Care placement
		Exposure to war/conflict
		Natural disasters
		Societal insecurity
		Sexually transmitted disease
		Homelessness

### Selection criteria

This review was conducted following PRISMA guidelines (Moher et al., 2009). A systematic database search was carried out on 16th March 2020 covering studies published up to the beginning of March 2020. Subsequently, another search was carried out on 6th October 2020 to capture additional studies released between the original search and completion of the original search while synthesis was ongoing. The databases searched were Scopus, MEDLINE via Ovid, PsycINFO via Ovid and Web of Science (Core Collection). Strings were devised thematically based on adversity, study design and the mediating or moderating relationships using Boolean search terms (see Table 2); each conceptual string was combined with OR and separate strings combined with AND. These strings were modified into Medical Subject Headings (MeSH) when searching in Ovid databases (see Supplementary Appendix A for detailed search strategies). In April 2020, the websites of the following cohort studies were directly searched for relevant studies: Longitudinal Study of Child Abuse and Neglect (LONGSCAN), The Dunedin Multidisciplinary Health and Development Study, E-Risk Longitudinal Twin Study, Avon Longitudinal Study of Parents and Children, 1958 National Child Development Study, British Cohort 1970, Context of Violence in Adolescence Cohort, Growing Up in Scotland, National Survey on Child and Adolescent Well-being, National Survey of Children’s Exposure to Violence, CDC-Kaiser Permanente Adverse Childhood Experiences Study, Christchurch Health and Development Study, National Epidemiologic Survey on Alcohol and Related Conditions and the Longitudinal Study of Australian Children.

**Table 2.** Boolean search terms used in systematic review.

Concept	Terms Used
Adversity/ACEs	child* adversity*, “adverse childhood experienc*”, child* trauma*, child* maltreat*, child* victimi*, child* abus*, “cumulative risk”
Study design	longitud*, prospect*, “cohort study”
Mechanism	moderat*, mediat*, mechanism*, pathway*, indirec*, interact*, resilien*

**Figure 1.**
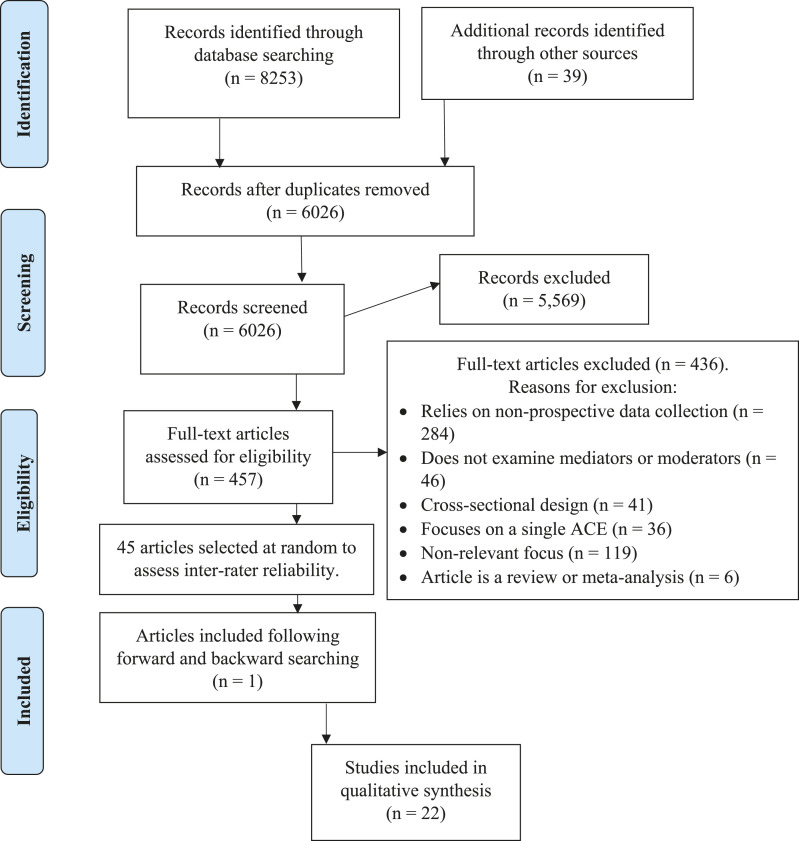
PRISMA diagram adapted from Moher et al. (2009).

Titles and abstracts of each article were screened, and those that seemed relevant were retrieved so the full-text article could be screened. Reference lists of included studies and studies that cited included studies were assessed for inclusion. Variables relating to study design, sample populations and findings were extracted. The process of the search strategy is displayed in Figure 1. The criteria that were used to include studies for the systematic review are found below. Based on the criteria, two raters (GH and ZES) assessed a random sample of 10% (45) full-text articles to represent the number of articles included. These 45 articles were sampled from the 457 full-text articles using a random number generator to represent the number of articles assessed for inclusion in the final review. There was a raw agreement of 91% between raters. Disagreements were ultimately settled to arrive at unanimous decisions, indicating good reliability of inclusion criteria.

A. Published in English, undergone peer review.
B. Utilised quantitative, prospective design that assessed the effect of mediating and moderating variables on the relationship between childhood adversity and outcomes in adulthood. There must have been at least two time points of data collection, where adversities were measured prior to outcomes.
C. Measured adversities including the following examples or related other adverse life circumstances: child abuse and neglect, witnessing domestic violence, witnessing crimes, criminal victimisation, exposure to community violence/war/terror, bullying, household dysfunction (e.g. substance use or mental illness in the household) and parent factors (e.g. incarcerated, deceased, separated or divorced).
D. Measured multiple (at least two) self-reported ACEs experienced by children (i.e. age lower than 19 years of age) or household dysfunction adversities either self-reported or reported by informants. Studies that relied only on official records of child maltreatment or retrospective measurement of adversities at age 19 and older were excluded.
E. Outcomes measured were related to adult mental health, physical health or life adjustment. Only studies assessing outcomes of participants over the age of 18 were included. Where a study sample represented age groups crossing the age of 18 (e.g. 16–20), the study was excluded unless results were separated for adults and adolescents.

## Results

### Study characteristics

See Table 3 for an overview of the characteristics and results of the 22 reviewed studies. The articles under review were published between 2006 and 2020. Notably, all but one study, which was conducted in the Netherlands (Veldman et al., 2015), were conducted in English-speaking countries including USA (*n* = 7), the UK (*n* = 6), Canada (*n* = 3), New Zealand (*n* = 2) and Australia (*n* = 4). The type of sample used for analysis varied, with birth cohorts (*n* = 13), school-age community (*n* = 4), high-risk for ACEs (*n* = 3) and juvenile delinquent or problem behaviour (*n* = 2) samples were used. Two samples recruited based on sex, with one female only sample and one male only sample.

**Table 3.** Table of studies included in systematic review.

Authors and Date	Dataset (Country)	Sample Characteristics	Number of Time Points	Number and Type of ACEs	Mediators and Moderators	Outcome	Findings
Bell et al. (2019)	Christchurch Health and Development Study (New Zealand)	A birth cohort of individuals born in the Christchurch region in 1977 (*N* = 962)	8	2–child maltreatment	Major depression, anxiety disorder, nicotine dependence, other illicit substance dependence and life stress	Psychotic experiences at age 30–35	Partially mediated
Byrd et al. (2019)	Pittsburgh Girls Study (USA)	Female sample of 5- to 8-year olds in Pittsburgh, oversampled low-income households (*N* = 2004)	16	4–child maltreatment 1–other	Emotional reactivity, MAOA genotype	Antisocial personality disorder and borderline personality disorder	Partial mediation through emotional reactivity, moderation through MAOA
Chen & Lacey (2018)	National Development Study (UK)	A birth cohort of individuals born in Great Britain during a 1-week period in 1958 (*N* = 7464)	9	1–child maltreatment 4–household dysfunction 1–other	Educational attainment, social class, emotional and somatic symptoms, problem alcohol use, smoking, physical exercise and BMI	Inflammation (CRP, fibrinogen and vWF) at age 44–45	Partial mediation through socioeconomic and health factors, but not psychological distress factors
Clark et al. (2010)	National Development Study (UK)	A birth cohort of individuals born in Great Britain during a 1-week period in 1958 (*N* = 9377)	9	3–child maltreatment 3–household dysfunction 1–other	Social class, father’s social class, housing tenure, sex and psychopathology^a^	Psychopathology at ages 23 and 45 (anxiety, affective and mood symptoms)	Mediation through early adult psychopathology
Dion et al. (2019)	N/A (Canada)	Students recruited from high schools (*N* = 370)	2	4–child maltreatment 1–household dysfunction	Attachment anxiety and attachment avoidance	Psychological distress and self-esteem at age 24	Mediation through attachment anxiety
Dubowitz et al. (2021)	LONGSCAN (USA)	A consortium of five American prospective studies (*N* = 473)	8	4–child maltreatment	Internalising or externalising problems	Alcohol and marijuana use at age 18	Mediation through internalising problems
Fergusson et al. (2011)	Christchurch Health and Development Study (New Zealand)	A birth cohort of individuals born in the Christchurch region in 1977 (*N* = 398)	8	2–child maltreatment 1–household dysfunction	MAOA (low-activity vs high-activity)	Hostility at ages 18, 21 and 25	MAOA moderated
Heinze et al. (2018)	N/A (USA)	Ninth-grade students recruited from high schools in Michigan (*N* = 676)	13	1–household dysfunction 2–other	Friendship attachment style (secure vs insecure)	Depression and anxiety growth trajectories between 19 and 32	Secure attachment to friends moderated
Huizinga et al. (2006)	National Youth Survey Family Study (USA)	Individuals aged 11–17 recruited to a national representative household study. Male sample (*N* = 1007)	9	1–child maltreatment 1–other	MAOA (high risk vs low risk)	Arrest records as an adult, antisocial personality disorder at ages 24–28	No moderation
Kelly-Irving, Lepage, Dedieu, Bartley, et al. (2013)	National Development Study (UK)	A birth cohort of individuals born in Great Britain during a 1-week period in 1958 (*N* = 15221)	9	1–child maltreatment 4–household dysfunction 1–other	Educational attainment, social class, depression, alcohol consumption, smoking status and BMI.	All-cause mortality	Partial mediation. More pronounced in male sub-sample than female sub-sample
Kelly-Irving, Lepage, Dedieu, Lacey, et al. (2013)	National Development Study (UK)	A birth cohort of individuals born in Great Britain during a 1-week period in 1958 (*N* = 6138)	9	1–child maltreatment 4–household dysfunction 1–other	Educational attainment, social class, depression, alcohol consumption, smoking status and BMI. For women, there was an additional mediator of having a first pregnancy prior to age 33	Cancer between ages 33 and 50	Partial mediation in female sub-sample, but no relationship in male sample
Miller et al. (2014)	LONGSCAN (USA)	A consortium of five American prospective studies (*N* = 884)	8	3–child maltreatment	Quality of relationships with parents, quality of friendships, depression	Suicidal ideation at age 18	Partial mediation through depression
Miller et al. (2018)	N/A (USA)	Two community samples of families with children aged 9–10 (*N* = 82)	6	4–Child maltreatment 5–household dysfunction 1–other	Cortisol awakening response	BMI at age 22	Full mediation through cortisol awakening response
Moretti & Craig (2013)	N/A (Canada)	Youths with either serious behaviour problems or involved in justice system (*N* = 179)	3	2–child maltreatment	Emotion dysregulation	Depressive symptoms at age 19	Partial mediation in male sample
Raposa, Bower, et al., 2014	Mater-University Queensland Study of Pregnancy (Australia)	A birth cohort study of mother-child dyads with depressed mother (*N* = 389)	6	1–child maltreatment 4–household dysfunction	Smoking, alcohol use, BMI, depressive symptoms and chronic stress in adulthood	Inflammation (CRP and sTNF-RII) between ages 22 and 25	Partial mediation through smoking status, BMI and chronic stress
Raposa, Hammen, et al. (2014)	Mater-University Queensland Study of Pregnancy (Australia)	A birth cohort study of mother-child dyads with depressed mother (*N* = 705)	6	1–child maltreatment 4–household dysfunction	Social stress, non-social stress depressive symptoms	Physical health (interviewer-rated, SF-16, chronic illnesses) at age 20	Mediation through depression and non-social stress
Raposa et al. (2015)	Mater-University Queensland Study of Pregnancy (Australia)	A birth cohort study of mother-child dyads with depressed mother (*N* = 175)	6	1–child maltreatment 4–household dysfunction	Presence of psychopathology in close friend	Depressive symptoms at ages 22–25	Mediation through the presence of a close friend with psychopathology symptoms
Schurer et al. (2019)	National Development Study (UK)	A birth cohort of individuals born in Great Britain during a 1-week period in 1958 (*N* = 7450)	9	1–child maltreatment 4–household dysfunction 1–other	Capital accumulated, cognitive skills, non-cognitive skills, mental and physical health, education attainment, family formation and labour force attachment	Economic outcomes (Net income, welfare dependence, subjective poverty) at age 55	Mediation through education attainment, cognitive skills, non-cognitive skills, health and family factors
Solís et al. (2015)	National Development Study (UK)	A birth cohort of individuals born in Great Britain during a 1-week period in 1958. Sample split by sex. *N* = 3753 men, *N* = 3782 women	9	1–child maltreatment 4–household dysfunction 1–other	Educational attainment, social class, socioeconomic status, marital status, physical activity, alcohol consumption, smoking, BMI and depressive symptoms	Allostatic load at age 44	Mediation through health factors, education level and wealth male sub-sample. Mediation through by health factors and being a homeowner at age 33 in female sub-sample
Starr et al. (2014)	Mater-University of Queensland Study of Pregnancy (Australia)	A birth cohort study of mother–child dyads with depressed mother. Sample split by available data for genotypic polymorphism (*N* = 705)	6	3–household dysfunction 1–other	Chronic stress in early adulthood. CRHR1 (rs110402 SNP) and 5-HTTLPR genotype	Depressive symptoms at age 20	Moderation by chronic stress and CRHR1. Moderated by three-way interaction of chronic stress, CRHR1 and 5-HTTLPR
Veldman et al. (2015)	Tracking Adolescents’ Individuals Lives Study (Netherlands)	A cohort study of children from 5 municipalities (*N* = 1881)	4	5–household dysfunction 3–other	Internalising problems, externalising problems and attention problems	Educational attainment at age 19	Partial mediation through externalising symptoms in male sub-sample
Wojciechowski (2020)	Pathways to Desistance (USA)	Juvenile offenders recently adjudicated for a serious offence (*N* = 261)	12	2–other	Anxiety trajectory (low, moderate and high)	Drug or alcohol dependence	Partial mediation through high trajectories of anxiety

^a^*Note.* This is not strictly a mediator because it is the same variable as measured as the outcome.

### Study designs

The age of participants at baseline ranged from at birth (*n* = 11, Bell et al., 2019; Chen & Lacey, 2018; Clark et al., 2010; Kelly-Irving, Lepage, Dedieu, Bartley, et al., 2013, Kelly-Irving, Lepage, Dedieu, Lacey, et al., 2013; Fergusson et al., 2011; Raposa, Bower, et al., 2014; Raposa, Hammen, 2014, 2015; Schurer et al., 2019; Solís et al., 2015; Starr et al., 2014) to age 11–17 (Huizinga et al., 2006). The age of participants at outcome measure ranged from 19 (Veldman et al., 2015) to 55 (Chen & Lacey, 2018; Clark et al., 2010; Kelly-Irving, Lepage, Dedieu, Bartley, et al., 2013; Kelly-Irving, Lepage, Dedieu, Lacey, et al., 2013; Schurer et al., 2019; Solís et al., 2015). The length of follow-ups varied considerably from 5 years to 55 years (M = 26.23, SD = 15.68). None of the assessed study designs formulated a CLPM to test longitudinal mediation. Sample sizes ranged from 82 to 15,221 (M = 2924.36, SD = 4080.52), indicating varying levels of statistical power amongst included studies. Characteristics of samples also varied, with 59% using general population samples (*n* = 13), 31.8% using at-risk samples (*n* = 7) and 9% using forensic/juvenile justice samples (*n* = 2).

Most of the included articles used secondary data from established cohort studies (*n* = 18), whereas a minority collected primary data (*n* = 4). The cohort studies that were used by articles included in this review were Christchurch Health and Development Study (*n* = 2), Pittsburgh Girls Study (*n* = 1), National Development Study (*n* = 6), LONGSCAN (*n* = 2), National Youth Survey Family Study (*n* = 1), Mater-University of Queensland Study of Pregnancy (*n* = 4), Tracking Adolescents’ Individuals Lives Study (*n* = 1) and Pathways to Desistance Study (*n* = 1). There was considerable overlap in the use of variables for studies using the National Development Study dataset, as well as studies that used the Mater-University of Queensland Study of Pregnancy dataset.

The combination of ACEs measured in included articles ranged from measuring two types of maltreatment and testing putative mediators separately (Bell et al., 2019) to measuring 10 ACEs from both child maltreatment and household dysfunction and other sub-categories and testing the putative mediators underlying a dose–response relationship (Miller et al., 2018). Seven studies measured fewer than four ACEs, limiting the ability to assess mediators and moderators of dose–response relationships with negative outcomes. The types of ACEs measured in included studies are shown in Table 1.

Throughout included studies, various terms are used to describe the general concept of ACEs, including child abuse, child maltreatment, abuse exposure, exposure to violence, childhood adversity, early life stress, early life adversity and poly-victimisation. There was much variation in how ACEs were measured from study to study, with most studies adopting a mixture of binary items that are either summed to create a composite or entered as individual variables (*n* = 15, Chen & Lacey, 2018; Clark et al., 2010; Dion et al., 2019; Heinze et al., 2018; Huizinga et al., 2006; Kelly-Irving, Lepage, Dedieu, Bartley, et al., 2013; Kelly-Irving, Lepage, Dedieu, Lacey, et al., 2013; Miller et al., 2014; Raposa, Bower, et al., 2014; Raposa, Hammen, 2014, 2015; Schurer et al., 2019; Solís et al., 2015; Veldman et al., 2015; Wojciechowski, 2020). Some studies used validated scales for individual variables or the whole composite of ACEs, such as the Adverse Childhood Experiences Scale, the Conflict Tactics Scale, Parent-Child Relationships Scale, Abuse Questionnaire, Structured Clinical Interview, Dyadic Adjustment Scale (Byrd et al., 2019; Miller et al., 2018; Moretti & Craig, 2013; Starr et al., 2014). One study designed its own scales for each measure (Dubowitz et al., 2021). Two studies were unclear in how they measured ACEs, although from both, it seemed as though single item measures were used (Bell et al., 2019; Fergusson et al., 2011).

#### Types of mediators/moderators

Mediators and moderators examined in this review are heterogeneous, capturing a wide variety of factors that can influence adult adjustment in the context of adversity. The most common types can be categorised as in different pathways, such as biological, psychological, additional stressors, health, personal assets, social, and family pathways. In most studies (*n* = 17), mediators or moderators were assessed before the outcome; in two studies, at the same time as the outcome; in one study, genetic polymorphisms were measured after the outcome and in two studies, it was unclear. Frequently examined mediators and moderators are shown in Table 4.

**Table 4.** Frequent mediators/moderators assessed and measured outcomes.

Mediators	Studies (*n*)	Outcomes Measured
Depression symptoms	7	Psychotic experiences, all-cause mortality, cancer, suicidal ideation, inflammation, physical health and allostatic load
Smoking status	5	Psychotic experiences, inflammation, all-cause mortality, cancer and allostatic load
Alcohol consumption	5	Inflammation, all-cause mortality, cancer and allostatic load
Body mass index	5	Cancer, inflammation and allostatic load
Educational attainment	5	Inflammation, all-cause mortality, cancer, economic success and allostatic load
Social class	5	Inflammation, psychopathology, all-cause mortality, cancer and allostatic load
Moderators		
MAOA genotype	3	Antisocial personality disorder, borderline personality disorder, hostility and criminality

*Note.* Only mediators and moderators assessed in three or more studies are presented in this table. The remaining mediators and moderators are sex, anxiety symptoms, anxiety growth trajectory, attachment type, emotional and somatic symptoms, psychopathology symptoms, internalising problems, externalising problems, attention problems, emotion regulation, emotional reactivity, presence of psychopathology in close friend, quality of friendships, quality of relationship with parents, life stress, social stress, non-social stress, chronic stress, mental and physical health, physical exercise, illicit substance use, cognitive skills, non-cognitive skills, capital accumulated, first pregnancy prior to age 33, family formation, housing tenure, marital status, parent’s social class, labour force attachment, cortisol awakening response, CRHR1 (rs110402 SNP) genotype and 5-HTTLPR genotype.

#### Psychopathology

Outcomes relevant to psychopathology include depression/mood symptoms (*n* = 5), anxiety symptoms (*n* = 2), antisocial personality disorder (*n* = 2), drug or alcohol dependence (*n* = 2) borderline personality disorder, psychotic experiences, suicidal ideation, self-esteem and general psychological distress. Evidence for mediating and moderating effects is mixed as few mediators and moderators are examined for similar outcomes across multiple studies. Most pathways tested were statistically significant. There was evidence that variables relevant to psychological distress or other psychopathology symptoms play an important role in the relationship between ACEs and later psychopathology symptoms (Bell et al., 2019; Clark et al., 2010; Dubowitz et al., 2021; Miller et al., 2014; Moretti & Craig, 2013; Wojciechowski, 2020). Some studies found that this was mediation via a different type of psychopathology symptom (Bell et al., 2019; Dubowitz et al., 2021; Moretti & Craig, 2013; Wojciechowski, 2020), whereas others were more of a continuity of symptoms (Clark et al., 2010; Miller et al., 2014). Specifically, depression and emotion dysregulation were mediators of subsequent psychotic experiences and depression symptoms, respectively (Bell et al., 2019; Moretti & Craig, 2013), and internalising problems including anxiety were mediators of drug or alcohol dependence (Dubowitz et al., 2021; Wojciechowski, 2020). There is also evidence that social factors (e.g. having a close friend with psychopathology symptoms) mediate the relationship between ACEs and mood disorder symptoms (Heinze et al., 2018; Raposa et al., 2015). Adolescent victimisation seemed to mediate between ACEs and antisocial personality disorder, but this was not moderated by monoamine oxidase A (MAOA) genotype (Huizinga et al., 2006). However, no two studies examined the same mechanism, so converging evidence is scant. For a full description of summarised results, see Table 3.

Out of 12 studies that assess the mediating and moderating variables in the relationship between ACEs and psychopathology, none appropriately accounted for stability of variance by repeating measures of independent variables, mediators and outcomes. Four out of 12 studies assessing psychopathology symptoms as an outcome accounted for a priori variance of similar symptoms at one of the previous time points. One study repeated measures of putative mediating and outcome variables at three sequential time points but did not do the same for ACEs (Moretti & Craig, 2013). One study controlled for substance use 2 years after baseline ACE measures and controlled for mediators at baseline (Dubowitz et al., 2021). Three studies controlled for the outcome measure at baseline (Clark et al., 2010; Dion et al., 2019; Miller et al., 2014), but one of these studies only employed a half-longitudinal design (Dion et al., 2019). Some studies utilised caregiver report when the participant was too young to self-report adversities (Raposa et al., 2015; Starr et al., 2014) or combined other methods of data collection alongside self-report (Bell et al., 2019; Clark et al., 2010; Dubowitz et al., 2021).

Several studies assessing psychopathology as an outcome studied sex differences, finding that some mechanisms may differ depending on sex. Two studies examined the interaction of MAOA genotype in the relationship between ACEs and personality disorders. Specifically, when male participants only were sampled, no moderation was found when the outcome was antisocial personality disorder (Huizinga et al., 2006). In a female only sample, high-activity MAOA genotype moderated the effect of ACEs on antisocial personality disorder and borderline personality disorder (Byrd et al., 2019). Specifically, high levels of ACEs and high-activity MAOA genotype increased the levels of emotion dysregulation, which subsequently predicted higher levels of personality disorder. Studies examining a sex interaction for psychological distress outcomes were mixed. When the outcome was suicidal ideation, one study found no sex interaction (Dion et al., 2019), whereas one study found that the mediation by social factors was stronger in a male sub-sample (Miller et al., 2014). When the outcome was depression symptoms, one study found no evidence of sex interaction (Raposa et al., 2015), another study found that sex was not a predictor of depression or anxiety growth trajectories (Heinze et al., 2018), and one study found that emotion dysregulation was a significant mediator only for the male sub-sample (Moretti & Craig, 2013). Finally, one study found no sex interaction in the relationship between ACEs and psychopathology symptoms (Clark et al., 2010). Taken together, these studies imply that sex is a moderator of the pathway between ACEs and personality disorders, but there is mixed evidence that sex differences are important for other psychopathological outcomes. Studies were limited in assessing differences based on ethnicity or socioeconomic status, although one study used an ethnically diverse sample (Miller et al., 2014).

#### Physical health

Of the studies that examining physical health outcomes, most found evidence for mechanistic pathways. Outcomes measuring mortality and physical health included inflammation (*n* = 2), mortality, cancer, body mass index, subjective physical health, chronic illness and allostatic load. Several studies found that health behaviours such as smoking status, physical exercise and body mass index were mediators of the relationship between ACEs and physical health outcomes (Chen & Lacey, 2018; Kelly-Irving, Lepage, Dedieu, Bartley, et al., 2013; Kelly-Irving, Lepage, Dedieu, Lacey, et al., 2013; Raposa, Bower, et al., 2014; Solís et al., 2015). Further, mixed findings indicated a mediation through socioeconomic factors (i.e. educational attainment and occupational social class; Chen & Lacey, 2018; Solís et al., 2015), and two found no mediation (Kelly-Irving, Lepage, Dedieu, Bartley, et al., 2013; Kelly-Irving, Lepage, Dedieu, Lacey, et al., 2013). However, all but two of these studies used the same dataset, the National Development Study. There is also tentative evidence that additional stressors contribute to health-related outcomes (Raposa, Bower, et al., 2014; Raposa, Hammen, 2014), but these two studies used the same dataset. For a full description of summarised results, see Table 3.

Out of seven studies that studied outcomes corresponding to physical health, all seven utilised several time points, but none repeated measures corresponding to the CLPM. All studies used a mixture of self-report and informants. Notably, six of the seven studies utilise two secondary datasets, the National Development Study (Chen & Lacey, 2018; Kelly-Irving, Lepage, Dedieu, Bartley, et al., 2013; Kelly-Irving, Lepage, Dedieu, Lacey, et al., 2013; Solís et al., 2015) and Mater-University Queensland Study of Pregnancy (Raposa, Bower, et al., 2014; Raposa, Hammen, 2014).

Several studies assessing physical health outcomes examined sex differences. Firstly, it was found that different mediators attenuated the relationship between ACEs and allostatic load (Solís et al., 2015). For men, health factors, education level and accumulated wealth mediated the relationship, whereas for women, health factors and being a homeowner at age 33 were mediators. Secondly, in the relationship between ACEs and mortality, the mediation effect was stronger for males than for females, with psychological malaise remaining a strong predictor of mortality even when mediators were added to the model (Kelly-Irving, Lepage, Dedieu, Bartley, et al., 2013). Thirdly, a study found no direct link between ACEs and cancer for males but did find a direct link for females (Kelly-Irving, Lepage, Dedieu, Lacey, et al., 2013). No sex interactions were found when the outcome was inflammation (Chen & Lacey, 2018). Studies were limited in assessing differences based on ethnicity.

#### Delinquency

Of the studies that examined delinquency as an outcome, both examined MAOA genotypes as moderators. Outcomes measured were hostility and arrest records. One study found that MAOA moderated the relationship between ACEs and hostility in early adulthood (Fergusson et al., 2011), whereas the other study found that MAOA did not moderate the relationship between ACEs and arrest records (Huizinga et al., 2006). This study also examined an interaction of sex, which was not significant (Huizinga et al., 2006). For a full description of summarised results, see Table 3. Both studies utilised several time points. Studies were limited in assessing differences based on ethnicity, although Fergusson et al. (2011) reported analyses both with and without ethnic minorities. In these separated analyses, the interaction effect was strengthened when ethnic minority data were omitted.

#### Personal achievements

Two studies examined mediating and moderating mechanisms in the relationship between ACEs and personal achievement. Both studies found evidence for mediating mechanisms such as cognitive skills, family formation, educational attainment and externalising problems (Schurer et al., 2019; Veldman et al., 2015). One study found that when stratified by sex, the model only remained significant for the male group (Veldman et al., 2015). For a full description of summarised results, see Table 3. Both studies utilised several time points, but neither study repeated measures corresponding to a CLPM. Both studies used a mixture of self-report and informant report in prospective design.

## Discussion

ACEs have been implicated in psychopathology, delinquency, poor physical health and poor socioeconomic outcomes. However, the general image of mediating and moderating effects is unclear based on the reviewed research. The main objective of this systematic review was to provide a synthesis of evidence regarding mediating and moderating mechanisms underlying the relationship between ACEs and negative outcomes in adulthood. The current review focused on prospective studies that used either self-report or informant report data of two or more ACEs.

In line with prior reviews which included cross-sectional studies (e.g. Gilbert et al., 2009; Hughes et al., 2017), the present review supported the basic longitudinal relationship between ACEs and multiple negative outcomes, particularly psychopathology and poor physical health. This review highlights some trends regarding the mediators underlying the relationship between ACEs and psychological distress. For instance, mediators relevant to psychological distress were found to be important in the relationship between ACEs and adult psychopathology. For depression, psychotic experiences, alcohol or drug dependence, suicidal ideation, mid-life psychopathology and self-esteem, at least one mediator was related to psychological distress (i.e. attachment anxiety, emotion dysregulation and sub-clinical distress), which might imply a predisposition, or an influence of stable environmental factors (see Hannigan et al., 2017). However, only one study investigated the influence of genotype on antisocial personality disorder and did not find evidence for moderation (Huizinga et al., 2006). Based on reviewed studies, earlier depression symptoms had the strongest evidence in several mediating psychological distress outcomes.

Regarding outcomes relevant to delinquency (such as hostility), genetic polymorphisms were assessed as moderators, but no mediators were assessed. Specifically, a high-activity MAOA genotype was found to moderate the relationships between ACEs and measures of hostility (Huizinga et al., 2006). A low-activity MAOA genotype was found to moderate the effect of ACEs on hostility, by increasing levels of hostility in early adulthood (Fergusson et al., 2011). There is relatively little to compare these findings to, as MAOA polymorphisms are most often assessed as risk factors for criminality (see Byrd & Manuck, 2014). For variables regarding physical health and early mortality, there was a trend for other health-related variables such as smoking status, body mass index, physical activity and alcohol consumption to partially mediate outcomes. This supports the findings of previous systematic reviews that relied on cross-sectional studies (Wiss & Brewerton, 2020). It is difficult to comment on the relative importance of each mediator, as reviewed studies tended to assess these together as ‘health factors’. To a lesser degree, variables related to socioeconomic conditions such as social class and education level, as well as depression partially mediated health outcomes.

The systematic review identified 22 prospective studies, which suggests that while ACEs are a popular research concept, the use of prospective longitudinal data to investigate mediation or moderation is uncommon. Included studies all adopted good study design features, but none adopted a longitudinal model ideally suited to infer mediating mechanisms. Crucially, most studies failed to repeat measures of independent, mediator and dependent variables over the course of the study, meaning conclusions often rely on untested assumptions (Preacher, 2015). One study compared the use of prospective self-report and retrospective self-report of child maltreatment and found considerable disagreement (Bell et al., 2019), emphasising the importance of deciding which data collection methods are most appropriate to measure ACEs. All included studies were published in the last 15 years, using data in English-speaking countries including USA, UK, Canada, New Zealand and Australia, with one exception being the Netherlands. Most samples represented the general population, while some at-risk and forensic populations were represented. A wide range of outcomes were assessed in these studies, such as psychopathology, mortality, delinquency, physical health, and educational or economic achievements. Similarly, a wide range of mediators and moderators were assessed, such as genotypic moderation, psychopathological symptoms, health behaviours and social conditions. Most studies tested several mediators or moderators simultaneously. However, because of the heterogeneity of mechanisms and outcomes addressed, a meta-analysis was not appropriate. Furthermore, the concept of ACEs was measured with great heterogeneity, with the range of ACEs studied being 2–10, and varying mixtures of child maltreatment, household dysfunction and other types of adversities.

### Limitations of the reviewed studies

The main limitation in reviewed studies is that the strength of study design was not ideally designed to test longitudinal mediation. Studies attempted to approximate a sequential design but were unable to account for potential longitudinal stability. Broadly, researchers should engage with literature regarding longitudinal panel modelling to use methods appropriate for testing underlying mechanisms, whether this be the CLPM or a different panel model (see Hamaker et al., 2015; Mund & Nestler, 2019). To increase certainty that outcome variance is due to a mediational mechanism observed in ACEs and the mediators in question, Preacher (2015) argues that there should be at least three time points at which independent, dependent and mediating variables are all measured. This allows researchers to control for a priori variance, which might confound the putative model. Only five studies attempted to control for prior variance of an outcome measure. For some outcomes, such as cancer and early mortality, controlling prior levels may not make conceptual sense, but controlling other well-documented risk factors, such as family history, may be worth consideration.

Another limitation of the present evidence base is that two large prospective studies account for 9 out of 22 (40.9%) of the reviewed papers: the National Development Study and the Mater-University Queensland Study of Pregnancy. Indubitably, these studies are useful to research questions concerning the longitudinal effects of childhood adversity. However, an over-reliance on two datasets means that the results synthesised may be unduly influenced by idiosyncrasies attributable to these datasets. It is appreciably difficult to obtain high-quality longitudinal data which assesses relevant variables. But it is important to ensure that findings can be generalised beyond popular datasets. More high-quality datasets that can be used to study longitudinal mechanisms are required.

One clear gap observed from the included articles is that despite the broad range of outcomes, disproportionate research attention focused on psychopathology. Only five of the outcomes measured appeared in more than one research article (depression, anxiety, antisocial personality disorder, inflammation and drug or alcohol dependence). To draw meaningful conclusions, the reviewed outcomes were subsumed into generic categories which may be arbitrary. Notably, while the original ACEs study found that ACEs were related to a plethora of leading causes of death (Felitti et al., 1998), none of the included studies assessed suicide attempts, sexually transmitted disease, diabetes, organ diseases or strokes. This omission belies several strong limitations of ACEs research, the reliance on a small number of datasets for longitudinal research and the general reliance on unreliable data collection methods (Widom et al., 2004). Specifically, many studies were excluded for relying solely on retrospective self-reports or court-substantiated records of child maltreatment. Only a handful of studies assessed positive outcome variables, substantially limiting the capacity of this review to synthesise knowledge about other pathways. To fully understand developmental processes tying ACEs to negative outcomes, it is important not to overlook normal developmental outcomes (Sroufe, 2013).

Another notable weakness of included studies is that most studies were comprised of ethnically and socioeconomically homogeneous samples. Some studies did investigate socioeconomic factors as mediators, which is important because low socioeconomic status tends to increase the risk of ACEs child maltreatment (Bywaters et al., 2017). There is some evidence that some ethnic minorities are more likely to be involved in child protection services, which indicates that ethnicity should be considered as a moderator (Putnam-Hornstein et al., 2013). Additionally, few studies examined sex as a moderator which further limits the insight as to relationships and mediated relationships dependent on sex. Considering that prevalence rates of ACEs seem to be influenced by the sex of the child (see Radford et al., 2011), it is also important to examine sex as a moderator.

### Recommendations for future studies

One way that future studies can improve is to ensure that study design is informed by longitudinal panel modelling designs appropriate to test underlying mechanisms. As a minimum, where researchers are interested in a mediating mechanism, study designs should enable researchers to control for variance over at least three time points. Failing to do so means that our conclusions rely on untested assumptions. Appropriate panel modelling techniques and suitable data will be most informative regarding developmental mechanisms (see Hamaker et al., 2015; Mund & Nestler, 2019; Preacher, 2015).

Secondly, research included in this systematic review tended to rely on a small number of prospective cohort studies. Equally, data assessed by studies included in this systematic review predominantly represented samples in USA, UK and Australia. Expanding on these samples is important for generalisability of study results. Research would benefit from new longitudinal data, and perhaps an increased focus on countries unrepresented by reviewed studies.

Thirdly, outcomes of interest to ACEs research vary from psychopathology, delinquency, physical health problems and economic output. However, research included in this review disproportionately studied psychopathological outcomes. Notably, none of the included studies investigated suicide attempts, sexually transmitted disease, diabetes, organ diseases or strokes as outcomes despite these being key outcomes in the original ACEs study (Felitti et al., 1998). Further research should seek to study the longitudinal mechanisms underlying the link between ACEs and outcomes that were not presented in this systematic review, as well as other important outcomes such as sleep disorders, criminality, and positive outcomes such as marriage, and economic success.

Fourthly, this systematic review captured a broad range of ACEs to reflect child maltreatment and household dysfunction, but several adverse experiences were not represented at all in this review. For instance, no studies measured exposure to war/conflict, societal insecurity, homelessness or natural disasters. This limits the research base of ACEs in representing adversity faced by children globally. Future studies could use data that measures such phenomena in a longitudinal manner alongside adversities such as child maltreatment or household dysfunction. The current global COVID-19 pandemic provides an opportunity to assess ACEs related to extraneous adversities. Indeed, prospective studies assessing ACEs related to the current pandemic should be set up now to further knowledge about the effect of ACEs.

Finally, there is a need to standardise the way that ACEs are measured in longitudinal research. Studies in this systematic review were sometimes measuring similar or identical concepts such as child abuse, child maltreatment, abuse exposure, exposure to violence, childhood adversity, early life stress, early life adversity and poly-victimisation. Arguably, these concepts encapsulate partial aspects of ACEs (Siddaway, 2020). There is a need to conceptually review ACEs with regard to assimilating similar or identical concepts into ACEs research to expand our understanding of how adversity affects outcomes in adulthood. Furthermore, there is a need for ACEs research to develop generalisable measures to enable better comparison between studies. From there, researchers can debate whether ACEs should be measured as individual variables, composite variables or other variations.

Recommendations for practice, policy and research are summarised in Table 5.

**Table 5.** Implications for practice, policy and research.

Practice
There is clear evidence of a relationship between ACEs and various negative outcomes in adulthood which supports previous cross-sectional data
Early occurrences of psychological distress and unhealthy behaviours are important in the relationship between ACEs and later adult psychological distress and poor health outcomes, respectively. Preventing long-term negative sequelae might necessitate intervention in adolescence or early adulthood for those with known histories of ACEs
Policy
Develop programs to prevent the longevity of psychological distress and unhealthy behaviours
Develop a commonly agreed upon definition of ACEs to improve comparison between studies and settings
Research
More research studying underlying mechanisms in relationship between ACEs and adult outcomes using prospective data needed. Theorised pathways should inform research design to aid the organisation of reviews and meta-analyses
Future study designs aiming to investigate mediating mechanisms should emulate a robust model that is able to account for stability of variance across multiple time points

### Limitations of this review

The present study should be interpreted in light of several limitations. Firstly, the review was limited to studies assessing adult outcomes, which means that it may have missed important prospective research regarding child and adolescent outcomes that could have been insightful. Additionally, the search strategy may have omitted relevant terms such as ‘potentially traumatic experiences’. This may mean that some relevant papers were missed in the search. We call on ACE researchers to converge on terminology to limit complexity in this research area. Secondly, this systematic review aimed to prioritise prospective self- and informant-report data which was justified by recent evidence that child maltreatment varies widely based on data collection method (see Baldwin et al., 2019; Newbury et al., 2018), and that prospective data has less reliance on life scripts and memory biases (see Widom et al., 2004). However, officially documented cases might be preferred due to greater certainty regarding the occurrence of ACEs. Our conclusions may differ due to our decision to focus on prospective self- or informant-report data, so it is imperative that future research investigates the effect of data collection methodology on putative mediation and moderation mechanisms underlying the relationship between ACEs and adult psychosocial functioning. Thirdly, only articles published in peer-reviewed journals were considered. Thus, the results synthesised are open to publication bias, especially in considering that most studies reported significant findings.

## Supplemental Material

sj-pdf-1-tva-10.1177_15248380221075087 – Supplemental Material for Links of Adversity in Childhood With Mental and Physical Health Outcomes: A Systematic Review of Longitudinal Mediating and Moderating Mechanisms Click here for additional data file. Supplemental Material, sj-pdf-1-tva-10.1177_15248380221075087 for Links of Adversity in Childhood With Mental and Physical Health Outcomes: A Systematic Review of Longitudinal Mediating and Moderating Mechanisms by George Kelvin Hales, Agata Debowska, Richard Rowe and Zeliha Ezgi Saribaz in Trauma, Violence, & Abuse
