# Supplementary material for: Links of Adversity in Childhood With Mental and Physical Health Outcomes: A Systematic Review of Longitudinal Mediating and Moderating Mechanisms
Source: Trauma Violence Abuse. 2022 Feb 28;24(3):1465–82. doi: 10.1177/15248380221075087 (PMC10240645; doi:10.1177/15248380221075087)
Supplement: sj-pdf-1-tva-10.1177_15248380221075087 – Supplemental Material for Links of Adversity in Childhood With Mental and Physical Health Outcomes: A Systematic Review of Longitudinal Mediating and Moderating Mechanisms [file sj-pdf-1-tva-10.1177_15248380221075087.pdf]

Appendix A

Search strategy

Scopus

(TITLE-ABS-KEY(child* adversity* OR “adverse childhood experienc*” OR child* trauma* OR child* maltreat* OR child* victimi* OR child* abus* OR “cumulative risk”) AND TITLE-ABS-KEY(longitud* OR prospect* OR “cohort study”) AND TITLE-ABS-KEY(moderat* OR mediat* OR mechanism* OR pathway OR indirec* OR resilen*))

Search screen: Advanced Search

Web of Science (Core Collection)

TOPIC: (child* adversity* OR "adverse childhood experienc*" OR child* trauma* OR child* maltreat* OR child* victimi* OR child* abus* OR "cumulative risk") *AND* **TOPIC:** (longitude* OR prospect* OR "cohort study") *AND* **TOPIC:** (moderat* OR mediat* OR mechanism* OR pathway OR indirec* OR resilen*)
Timespan: All years.
Databases: WOS.
Search language=English

Medline via Ovid

1 adverse childhood experience.mp. or exp Adverse Childhood Experiences/

2 child abuse.mp. or exp Child Abuse/

3 childhood trauma.mp.

4 child maltreatment.mp.

5 child victimisation.mp.

6 cumulative risk.mp.

7 exp Longitudinal Studies/ or longitudinal.mp.

8 prospective study.mp. or exp Prospective Studies/

9 cohort study.mp. or exp Cohort Studies/

10 moderation.mp.

11 mediation.mp.

12 mechanism.mp.

13 pathway.mp.

14 indirect.mp.

15 Resilience, Psychological/ or resilience.mp.

16 1 or 2 or 3 or 4 or 5 or 6

17 7 or 8 or 9

18 10 or 11 or 12 or 13 or 14 or 15

19 16 and 17 and 18

PsycINFO via Ovid

1 exp Childhood Adversity/ or exp Adversity/ or exp Child Abuse/ or child adversity.mp.

2 adverse childhood experiences.mp.

3 childhood trauma.mp.

4 exp Sexual Abuse/ or exp Victimization/ or childhood maltreatment.mp. or exp Child Neglect/

5 childhood victimisation.mp.

6 cumulative risk.mp.

7 exp Longitudinal Studies/ or longitudinal.mp.

8 exp Prospective Studies/ or prospective.mp.

9 cohort study.mp.

10 exp Mediation/ or moderation.mp.

11 mechanism.mp.

12 pathway.mp.

13	indirect.mp.
14	exp "Resilience (Psychological)"/ or resilience.mp.
15	1 or 2 or 3 or 4 or 5 or 6
16	7 or 8 or 9
17	10 or 11 or 12 or 13 or 14
18	15 and 16 and 17
